# The impact of diabetes mellitus on tendon pathology: a review

**DOI:** 10.3389/fphar.2024.1491633

**Published:** 2024-11-05

**Authors:** Jian Xu, Jinbo Wang, Yuncong Ji, Yanlong Liu, Jishi Jiang, Yanbo Wang, Xilong Cui, Yunpeng Wan, Biao Guo, Haiyang Yu

**Affiliations:** ^1^ Department of Orthopedics, Sports Medicine and Arthroscopy, Affiliated Fuyang People’s Hospital of Anhui Medical University, Fuyang, Anhui, China; ^2^ Shanghai Jiao Tong University School of Medicine, Shanghai, China

**Keywords:** diabetes, tendon, tendinopathy, pathology, fibrosis

## Abstract

Diabetes is one of the most common metabolic diseases worldwide, leading to complications, mortality, and significant healthcare expenditures, which impose a substantial social and financial burden globally. A diabetic environment can induce metabolic changes, negatively affecting tendon homeostasis, leading to alterations in biomechanical properties and histopathology. Numerous studies have investigated the mechanisms through which diabetes exerts pathological effects on tendons, including increased free radical production, oxidative stress, inflammatory responses, deposition of advanced glycation end products (AGEs), and microvascular changes. These metabolic changes damages tendon structure, biomechanics, and tendon repair processes. The proliferation of tendon stem cells decreases, apoptosis increases, and abnormal differentiation, along with abnormal expression of myofibroblasts, ultimately lead to insufficient tendon repair, fibrosis, and remodeling. Although researches unveiling the effects of diabetes on tendinopathy, fibrosis or contracture, and tendon injury healing are growing, systematic understanding is still lacking. Therefore, this review summarizes the current research status and provides a comprehensive overview, offering theoretical guidance for future in-depth exploration of the impact of diabetes on tendons and the development of treatments for diabetes-related tendon diseases.

## 1 Introduction

Diabetes mellitus (DM) is a metabolic disorder characterized by hyperglycemia, primarily caused by insufficient insulin secretion (type 1 diabetes, T1DM) or insulin resistance (type 2 diabetes, T2DM) (DiMeglio et al., 2018; Chatterjee et al., 2017). The latter accounts for approximately 90% of all diabetes cases and is one of the most prevalent metabolic diseases worldwide (Chatterjee et al., 2017; Giha et al., 2022). By 2045, the prevalence is projected to rise to 12.2% of the population worldwide (Sun et al., 2022). The high prevalence of DM has significant social, economic, and developmental implications (Chatterjee et al., 2017; Vasiljević et al., 2022). Complications, mortality, and healthcare costs associated with DM impose a considerable social and financial burden (Cho et al., 2018; Nichols et al., 2019).

DM induces metabolic changes in microenvironment, such as increased free radical production, oxidative stress, abnormal expression of inflammatory factors (Vasiljević et al., 2022), copper metabolism abnormalities (Jia et al., 2024a), and the deposition of advanced glycation end products (AGEs) (Lee and Veres, 2019; Haus et al., 2007) These diabetes-related microenvironmental changes lead to numerous clinical complications, such microvascular diseases (Puri et al., 2022) and macrovascular diseases (Puri et al., 2022; Kato et al., 2024). In addition, musculoskeletal abnormalities, including tendon dysfunction, are also common complications of diabetes (Giha et al., 2022; Shalit et al., 2024).

Tendons connect muscles and bones, effectively transmitting muscle forces during musculoskeletal movements (Lu et al., 2020; Sharma and Maffulli, 2005; Singh et al., 2022). While there are extensive research on the impact of DM on musculoskeletal disorders, including arthritis (Wang et al., 2024a; Banu and Köseoğlu, 2023), osteoporosis (Li et al., 2024a), skeletal muscle atrophy (Atala et al., 2021; Cruz-Jentoft et al., 2019), and fibrosis (Singh et al., 2022; Wu et al., 2024), recent years witnesses an increasing number of investigations on the effects of DM on tendon homeostasis, providing knowledgeable foundation for further studies.

## 2 Impact of DM on normal tendons

The primary components of tendons are dense fibrous connective tissue and collagen, connecting muscles to bones and efficiently transmits forces during movements (Lu et al., 2020; Sharma and Maffulli, 2005). T2DM leads to impaired cellular glucose uptake and chronic hyperglycemia, exposing tissues to abnormally high glucose concentrations (Chatterjee et al., 2017). In both basic and clinical studies, the impact of T2DM on tendon homeostasis is generally overlooked, possibly due to a lack of recognition of the chronic pathological changes in tendon structure caused by T2DM (Nichols et al., 2019; Kim et al., 2022; Filgueiras et al., 2022).

DM alters muscle microcirculation and metabolic responses. In diabetic patients with a high risk of peripheral arterial or neurological disease, microcirculation deterioration is present in muscles and tendons, and tendon homeostasis may be affected by hyperglycemia (Kim et al., 2022; Kim et al., 2021; Panji Sananta et al., 2019), leading to structural changes and inflammation (Nopparat et al., 2023). Furthermore, T2DM is associated with increased oxidative stress (OS), which negatively affects tendon conditions (Vasiljević et al., 2022; Atala et al., 2021; Alabadi et al., 2023). Advanced glycation end products (AGEs) are compounds formed by aging and DM, which activate NADPH oxidase (NOX), increase reactive oxygen species (ROS) production and leads to OS (Kato et al., 2023; Shinohara et al., 2022a). AGEs also induces OS and triggers inflammatory responses (Shinohara et al., 2022a). The accumulation of AGEs, combined with other systemic and behavioral factors, further complicates tendon dysfunction (Singh et al., 2022; Zellers et al., 2021). AGEs, formed by non-enzymatic reactions, bind to membrane receptors to exacerbate inflammation and accelerate protein degradation (Puri et al., 2022; Cruz-Jentoft et al., 2019).

Structural changes in tendons of DM patients include collagen fiber disorder and micro-tears (Lo et al., 2013; Zaib et al., 2024; Chang et al., 2022). The metabolic changes in the microenvironment affect tendon stiffness, collagen composition, and physiology (Lee and Veres, 2019; Shi et al., 2021), which may be associated with AGEs (Fessel et al., 2014; Li et al., 2013). Research has shown that the crosslinking of AGEs in DM tendon inhibits the biomechanical plasticity and significantly disrupts tissue morphology (Lee and Veres, 2019; Indyk et al., 2021). The accumulated AGEs not only crosslinks adjacent collagen molecules to weaken biomechanics (Lee and Veres, 2019), but also induces inflammatory responses (Indyk et al., 2021). Moreover, pro-inflammatory chemokines, such as CCL-1, 2, 4, and 5, are highly expressed in the circulation of T2DM, further mediating inflammation (Mir et al., 2024).

Degenerative changes in tendons are common in DM patients (Abate et al., 2010). For example, histological studies confirm that hyperglycemia caused by DM is associated with degeneration of the rotator cuff or Achilles tendons (Kim et al., 2022; Kent and Bailey, 1985). Even asymptomatic DM patients may exhibit morphological abnormalities in the Achilles tendon (Afolabi et al., 2020), such as thickening, collagen disorder, or calcific changes at the tendon-bone junction (Harish et al., 2020; Vaidya et al., 2022; Xu et al., 2022). Specifically, Sneha et al. (Harish et al., 2020) evaluated the Achilles tendons of 61 healthy volunteers and 81 T2DM patients using ultrasound. Compared with healthy volunteers, the Achilles in T2DM patients was thickened and softened. DM can also lead to increased risk of Achilles tendon and plantar fascia contracture, impairing foot biomechanics and contributing to foot ulcers (Zellers et al., 2021; Harish et al., 2020; Ra and Hn, 2022). These changes reduce the extensibility of normal tendons and the strain energy of rupture (Lopez-Pedrosa et al., 2024; Su et al., 2024).

## 3 Impact of DM on tendinopathy

**FIGURE 1:**
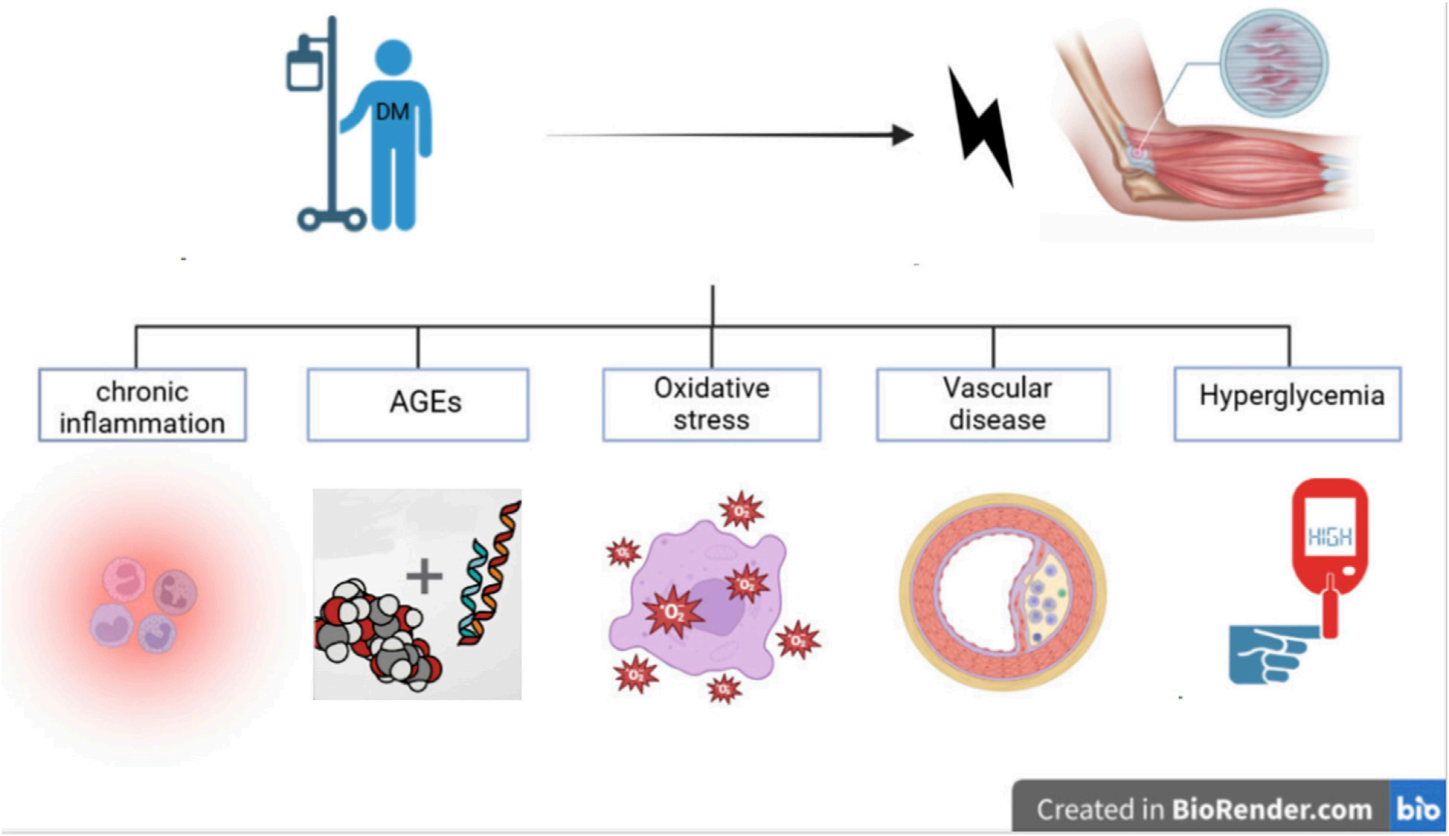
Schematic illustration of the potential mechanisms by whcich diabetes mellitus triggers tendinopathy.

Tendinopathy is a common connective tissue disease, widely described as involving cellular proliferation, changes in extracellular matrix (ECM) turnover/synthesis, and inflammation associated with chronic tendon pathology (Sikes et al., 2021). The etiology is multifactorial and not yet fully understood (Giha et al., 2022; Xu et al., 2022). Tendinopathy is usually caused by overuse, metabolic disorders, and other metabolic factors related to micro-injuries in tendons. Tendinopathy is a challenging complication in diabetic patients (Shi et al., 2021; Cannata et al., 2020), often leading to chronic pain, restricted joint mobility, and even tendon rupture. DM, especially hyperglycemia, leads to elevated levels of acetylated p53, promoting cell apoptosis and OS, shifting the response of tenocytes from anabolic to pathogenic (Chang et al., 2022; Shinohara et al., 2022b), increasing the risk of developing tendinopathy (Panji Sananta et al., 2019; Harish et al., 2020). The potential pathogenic mechanisms by which DM leads to tendinopathy can generally be categorized into several aspects (Figure 1).

Chronic Inflammation: It is well known that diabetic patients are in a pro-inflammatory state, and the hyperglycemic environment in diabetes may lead to chronic inflammation in tendons, eventually progressing to tendinopathy (Kwan et al., 2020). Diabetic patients typically exhibit elevated levels of pro-inflammatory cytokines, such as prostaglandins, tumor necrosis factor-α (TNF-α), interleukin-6 (IL-6), and leukotriene B4, which are significantly elevated in the serum of diabetic patients (Vasiljević et al., 2022; Zaib et al., 2024). These elevated levels of pro-inflammatory cytokines and chemokines may contribute to the chronic development of tendinopathy (Indyk et al., 2021; Xu et al., 2022; Kwan et al., 2020). Evidence indicates that the chronic inflammation observed in tendinopathy may be due to the reduced proteolytic response of tendon-derived stem cells (TDSCs) in tendinopathy, where the hyperglycemic environment may stimulate chronic inflammation and reduced proteolytic response, leading to tendinopathy (Kwan et al., 2020). Studies on the role of T2DM in rotator cuff tendinopathy suggest that persistent hyperglycemia may impair the proliferation and autophagy of tenocytes, further leading to increased expression of pro-inflammatory and pro-fibrotic mediators (Song et al., 2022).

Excessive Production of AGEs: AGEs can alter collagen within tendons, increase collagen crosslinking, reduce tendon fiber sliding and viscoelasticity, inhibit the biomechanical plasticity of natural tendons, and disrupt tendon morphology (Lee and Veres, 2019; Indyk et al., 2021). TDSCs are involved in tendinopathy, and AGEs can alter the pathophysiology of tendons in diabetic patients by regulating the proliferation and differentiation of TSPCs (Lu et al., 2020). However, other studies suggest that the relationship between AGE content and tendon tensile mechanics may be obscured by collagen disorder (Zellers et al., 2021).

OS: Diabetic patients may experience impaired angiogenesis, promoting tissue hypoxia and the production of ROS, leading to OS and pathological damage (Abu Khadra et al., 2024). In addition, DM patients have lower levels of catalase (CAT) activity, with an imbalance between oxidants and antioxidants, which increases OS to induce cell death and trigger tendinopathy (Lu et al., 2020; Abu Khadra et al., 2024; Yoon et al., 2024).

Vascular Changes: Vascular disease is one of the most common long-term complications of poorly DM, leading to functional and structural changes in the macrovascular and microvascular systems of tendons (Panji Sananta et al., 2019). These biochemical and structural abnormalities are also observed in various organs and tissues, including nephropathy, retinopathy, peripheral neuropathy, atherosclerosis, etc. (Kato et al., 2024; Tavares et al., 2021; Zheng et al., 2021). Diabetes-induced endothelial cell damage reduces the synthesis and secretion of protective factors, resulting in vasoconstriction and inflammation (Sharma and Maffulli, 2005).

Circulating AGEs are associated with vascular complications (Kato et al., 2024). Impaired vascular supply may also reduce the nutrients and oxygen supply to connective tissues, leading to degenerative changes and hindering tendon healing, thus promoting tendinopathy (Kato et al., 2024; Indyk et al., 2021; Abu Khadra et al., 2024). Some studies suggest that dysregulated glucose and lipid metabolism exacerbate the aging of TDSCs and promote osteogenic differentiation (Chen et al., 2024).

Calcific tendinopathy of the Achilles tendon is common, but most patients are asymptomatic. The incidence of Achilles tendon insertional calcific tendinopathy increases with age and is significantly higher in diabetic patients (Giai Via et al., 2022). Research shows that the risk of developing calcific tendinopathy of the shoulder increases by 27% at 8 years following DM diagnosis (Su et al., 2021). On the other hand, the etiology and pathogenesis of calcific tendinopathy remain unclear. Riley et al. (1994). proposed a theory suggesting that ischemic injury and rotator cuff degeneration associated with metabolic diseases lead to further calcification, indicating that metabolic diseases may be related to calcific tendinopathy. Chen et al. (2024) demonstrated that dysregulated glucose and lipid metabolism can activate the CXCL13-CXCR5 axis in aged TDSCs, thereby promoting ectopic ossification.

Hyperglycemia, inflammatory responses, AGEs, OS, and diabetic vascular changes can all influence tendon cell behavior. However, the extent to which these specific changes lead to diabetic tendinopathy and impaired healing remains unclear (Vaidya et al., 2022). Antidiabetic drugs may have beneficial effects on diabetic tendinopathy. Pioglitazone improves TDSC dysfunction caused by AGEs through autophagy promotion, and pioglitazone has been identified as a potential pharmacological option for tendinopathy (Xu et al., 2020). Research on metformin suggests that it may affect gene expression of myogenesis and adipogenesis, while whether metformin benefits tendinopathy remain unclear (Chang et al., 2022). Further efforts are required to develop effective therapeutics.

## 4 Impact of DM on tendon fibrosis

DM is associated with several fibrotic conditions, such as frozen shoulder, Dupuytren’s contracture, trigger finger, Achilles tendon contracture, and plantar fasciitis, which limit the range of motion of the affected joints, impairing function and the ability to perform daily activities (Abate et al., 2013; Al-Matubsi et al., 2011). Fibrosis is characterized by the accumulation of ECM, usually involving changes in ECM quality. The morphological and biochemical disruption of the ECM is directly related to the loss of target organ function (Primadhi and Herman, 2021; Ramirez et al., 2024). The excessive production of AGEs under hyperglycemic conditions can alter collagen within tendons, increase collagen crosslinking, reduce tendon fiber sliding and viscoelasticity, inhibit the biomechanical plasticity of natural tendons, and disrupt tendon morphology (Lee and Veres, 2019; Indyk et al., 2021; Gautieri and Silván, 2016). By stimulating transforming growth factor-beta (TGF-β) pathway, AGEs and ROS regulate the expression of various matrix proteins, forming fibrotic tissue (Primadhi and Herman, 2021; Li et al., 2024b; Noonin and Thongboonkerd, 2024). Myofibroblasts, the main producers and organizers of collagen/ECM during tissue healing, are also sensitive to DM related pathological changes, initiating hypertrophic scar formation and tissue fibrosis (Schuster et al., 2023). Given the aberrant fibrogenesis process, T2DM significantly impairs tendon healing by inducing scar formation (Zhao et al., 2017).

Tendon injuries can occur at the muscle-tendon junction (e.g., gastrocnemius, quadriceps), within the tendon itself (e.g., Achilles tendon), and at the tendon-bone interface (e.g., rotator cuff) (Sharma and Maffulli, 2005; Tavares et al., 2021; Takahashi et al., 2021; Yuan et al., 2024). Tendon healing occurs in three overlapping phases: the initial inflammatory phase, where erythrocytes and inflammatory cells, particularly neutrophils, infiltrate the injury site, with monocytes and macrophages predominating within the first 24 h, leading to the phagocytosis of necrotic material; a few days later, the proliferative phase begins and lasts for several weeks, during which the synthesis of type III collagen peaks; approximately 6 weeks later, the remodeling phase begins, characterized by a reduction in cell numbers, and decreased collagen and glycosaminoglycan synthesis. The remodeling phase can be divided into a consolidation phase, beginning around 6 weeks and lasting up to 10 weeks, and a maturation phase, starting 10 weeks after injury and continuing for up to a year, during which fibrous tissue gradually transforms into scar-like tendon tissue (Sharma and Maffulli, 2005; Farkas et al., 1973; Adawhhflf et al., 1983).

The increased risk of rotator cuff tears (RCTs) in diabetic patients may be related to impaired microcirculation (Yuan et al., 2024). Studies have shown that sodium-glucose cotransporter 2 inhibitors (SGLT2is) promote systemic anti-inflammatory effects by increasing fat utilization and regulating macrophage-mediated inflammatory pathways. SGLT2 inhibitors may prevent rotator cuff tears and subsequent repairs by reducing inflammation (Su et al., 2024). Diabetes leads to severe damage to the inflammatory, angiogenic, and proliferative processes, which may adversely affect tendon healing or remodeling after injury (Chbinou and Frenette, 2004).

Diabetic patients are at a higher risk of requiring tendon repair surgery (Cho et al., 2015), and diabetes can affect tendon healing post-operatively (Tavares et al., 2021; Takahashi et al., 2021; Griffith et al., 2022). Elevated hemoglobin A1c levels 3–6 months after rotator cuff repair surgery in diabetic patients are associated with an increased rate of re-tears (Kim et al., 2023). Nevertheless, for diabetic patients with perioperative glycemic control, the re-tear rate following rotator cuff repair is observed to be comparable to that of non-diabetic patients (Smith et al., 2021), underlying the importance of blood glucose control.

Tendon-bone healing is a challenging process in orthopedics and sports medicine (Wang et al., 2024b), while DM is a significant risk factor for poor tendon-to-bone healing. The hyperglycemic microenvironment inhibits TDSCs proliferation and inducing osteochondral differentiation, a potential mechanism by which diabetes impairs tendon-to-bone healing (Cao et al., 2022). Additionally, diabetes-induced hyperglycemia increases the expression of AGE and RAGE, resulting in significantly elevated mRNA expression levels of NOX1, NOX4, IL-6, RAGE, type III collagen, MMP2, TIMP1, and TIMP2 in the rotator cuff tendon, along with an increase in ROS-positive cells and apoptotic cells (Lee and Veres, 2019; Shinohara et al., 2022b; Yoshikawa et al., 2022). These inflammatory factors also induce a crosstalk between immune cells and tenocytes/TDSCs, while breaking this vicious cycle has therapeutic potential against this condition (Peng et al., 2024). Fibroblasts is closely correlated with collagen levels, and a hyperglycemic environment negatively impacts fibroblast quantity, adversely affecting tendon healing (Panji Sananta et al., 2019). AGEs-related increased expression of inflammatory factors can result in insufficient type I collagen synthesis of fibroblasts, delaying recovery process (Yoshikawa et al., 2022; Jia et al., 2024b).

## 5 Potential therapies under development

The ability to manage targets related to tendinopathy/tendon healing and strictly control diabetes may be effective in treating tendon pathology in diabetic patients (Yoon et al., 2024). However, the cellular and molecular components involved in various aspects of tendons disrupted by diabetes remain to be elucidated (Yoon et al., 2024). AGE inhibitors that prevent AGE formation could be a novel approach to treating diabetic tendon-to-bone healing (Jud and Sourij, 2019) (Menè and Pugliese, 2003). These therapeutic options include AGE crosslink breakers, AGE inhibitors, RAGE antagonists, clinically approved drugs for various indications (e.g., antidiabetic and antihypertensive drugs, or statins), and dietary and herbal treatments (Jud and Sourij, 2019). Direct AGE inhibitors include pyridoxamine and aminoguanidine, which reduce AGE/RAGE by increasing activation of the detoxifying enzyme Glo-1 and inhibiting ROS derived from NOX, as well as by inhibiting the formation of reactive dicarbonyl compounds (such as methylglyoxal) (Sourris et al., 2020). Hyperglycemic conditions increase intracellular ROS levels, a major cause of OS, which may interfere with the repair capacity of damaged or degenerated tendons under hyperglycemic conditions (Yoon et al., 2024; Osonoi et al., 2020). Inhibiting OS and improving mitochondrial function is another manner to facilitate tissue repair (Li et al., 2024c; Quetglas-Llabrés et al., 2024). Dietary polyphenols is noticed to mitigate OS and mitochondrial dysfunction in the crosstalk between type 2 diabetes and tendinopathy (Wang et al., 2024c). Polyphenols, such as pomegranate peel extract, have also shown beneficial effects on inflammatory states and OS biomarkers in T2DM (Vasiljević et al., 2022).

The decline in regenerative function of adipose-derived stem cells is partly mediated by the OS and inflammatory environment induced by diabetes. The induction of antioxidant stress factors in adipose-derived stem cells may represent an adaptive mechanism to cope with the increased OS in the diabetic microenvironment (Ahmed et al., 2024). After applying adipose tissue-derived stromal vascular fraction (SVF) in diabetic rats, the number of tenocytes, capillaries, and collagen increased, improving Achilles tendon rupture healing (Panji Sananta et al., 2019). 3D-printed biological scaffolds have the potential to improve rotator cuff healing by enhancing osteogenesis, reducing inflammation, and promoting macrophage polarization (Wang et al., 2024b). Some studies also suggest that antidiabetic drugs may have beneficial effects on tendon healing. For example, pioglitazone can prevent the harmful effects of AGEs on Achilles tendon healing, improving the biomechanical properties of the Achilles tendon (Jia et al., 2024b). Pioglitazone is a peroxisome proliferator-activated receptor-gamma (PPAR-γ) agonist widely used in clinical practice to treat T2DM. It can also reduce RAGE expression and block its downstream signaling pathways, thereby alleviating OS and inflammation in tissues (Xu et al., 2020; Yuan et al., 2011). Diabetes has adverse effects on the neurotrophic pathways in tendon regeneration. Therefore, new therapeutic strategies for regenerating tendons after injury in diabetic patients may include the modulation of neurotrophic pathway molecules, such as NGF and its receptors (Quaini et al., 2017).

## 6 Conclusion

In summary, DM alters the microcirculation and metabolic responses in tendons, leading to negative changes that affect the biomechanical properties and histopathology. Specifically, increased free radical production, OS, inflammatory responses, and the deposition of AGEs collectively damage tendon structure, biomechanics, and tendon fibrosis and repair. The decreased proliferation of tendon stem cells, increased apoptosis, and incorrect differentiation ultimately result in insufficient tendon repair, maintenance, and remodeling. Although current research has explored the impact of diabetes on tendons, tendinopathy, and tendon injury healing, detailed evidence on the underlying mechanisms remains to be revealed. Future researches are needed to delve deeper into the mechanisms DM-associated tendon pathology to provide references for developing treatment methods against this disorder.
